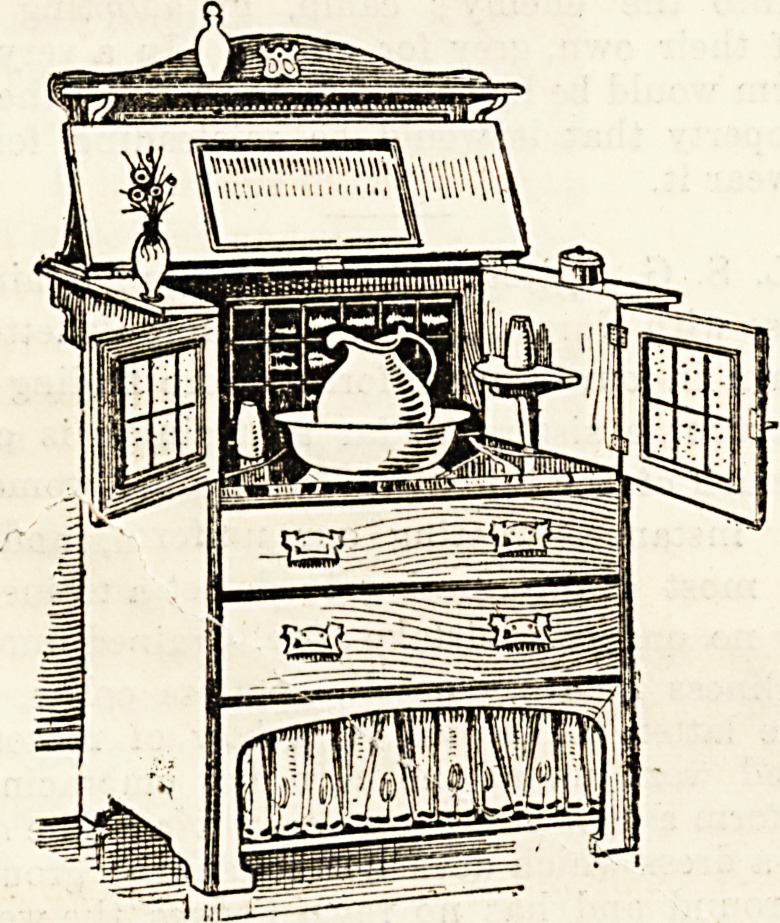# The Hospital. Nursing Section

**Published:** 1905-04-29

**Authors:** 


### The Hospital.

### Nursing Section.

Contributions for this Section of "The Hospital" should be addressed to the Editor, "The Hospital" Nursing Section, 28 & 29 Southampton Street, Strand, London, W.C.

No. 970.—Vol. XXXVIII. SATURDAY, APRIL 29, 1905.

### Notes on News from the Nursing World.

### PARLIAMENT AND THE REGISTRATION BILLS.

The Nurses and Private Nursing Home Registration Bill is now down for second reading in the House of Commons on Monday, May 8, the fourth order on a Government night. The rival measure, the Nurses' Registration Bill, stands second of private measures on Tuesday, May 9. In addition to Mr. Harry Lawson, Mr. M'Kenna has given notice of his intention to move the rejection of the latter Bill.

### COLONIAL SISTERS AND AMATEURS.

An extraordinary situation has arisen in Hongkong. A short time ago it was decided to start nursing classes for the benefit of some of the European ladies in the colony, and it appears that the authorities of the Civil Hospital require the sisters to give instruction gratuitously on these occasions. The sisters object to this so strongly that there is talk of a strike among them. We hope that they will not be so ill-advised as to resort to action which it is almost impossible under any circumstances to justify. But they have a perfect right to protest against being saddled with work which has nothing to do with the hospital, either in their leisure or at any other time. If the European ladies in Hongkong desire to get a smattering of nursing, which is more likely to do them harm than good, they should be prepared to pay their instructors properly.

### BOMBAY HOSPITAL NURSES.

At the annual meeting of the subscribers to the St. George's Hospital Nursing Association, Bombay, the report of the Committee was submitted. The most important features referred to the formation of the new Provident Fund and a scheme for the amalgamation of the Bombay Nursing Associations into a general nursing service for the Presidency. As to the former, the report states that the Committee have transferred all the surplus assets belonging to the old Provident Fund to the credit of the Reserve Fund Account; and, as to the latter, the amalgamation is at present receiving the consideration of the Government, who have appointed a joint committee under the chairmanship of the Surgeon-General to go into the question and to report the lines upon which such a service, if at all practicable, could be worked. It is noteworthy that, owing to the increasing demand for private nurses, the Committee were able in December last to reduce the charge to the former rate of 5 rupees per day for ordinary cases. The report from the physician in charge speaks well of the nursing in the wards of the hospital. With the consent of the Government there has been instituted an annual course of lectures on anatomy, physiology, and surgery, for the instruction of nurses going up for the examination to qualify them as seniors. The medical man who delivers the lectures gave a prize last year to the nurse who passed highest in this examination, which was won by Miss Charlotte Mullen.

### COREAN BOYS AS NURSES.

The account sent to us of the Mission Hospital at Chemulpo, which has virtually been rebuilt by Mrs. Weir, the lady superintendent, who was trained at St. Bartholomew's Hospital, where she won the gold medal, will be read with the greatest interest. It shows that even in the hermit kingdom there is steady progress, and that Corean women, who are not supposed to see any man except their nearest relatives, attend the hospital for treatment. The great and immediate want, it will be seen, is a trained nurse, though the two Corean boys who are employed in assisting to nurse and discharge other duties, acquit themselves well under the circumstances. Perhaps when the lady superintendent obtains the help of the nurse she so much needs it may become practicable to provide the small female ward which is likely to be increasingly wanted.

### WORKHOUSE NURSING AND A REPORT.

There are several unsatisfactory features in the fourteenth annual report of the Northern Workhouse Nursing Association. One is the statement that there are still boards of guardians who do not hesitate to appoint absolutely untrained persons as nurses in their infirmaries, though this, of course, is not news. It can hardly be a matter for congratulation that during the year 33 applications for nurses were received by the Association, and 15 appointments made. The report anticipates the criticism that these figures seem small when compared with the total number of nurses employed in Poor-law infirmaries. Mention is also made of the case of one union in which the conditions were such that the committee felt compelled to remove the Association nurses, and decided to send no others to the service of the board until improvements had been made Yet another unsatisfactory feature is the fact that the expenses of the Association for the year exceeded the receipts to the extent of £72. We observe that the report attributes "the unpopularity of workhouse nursing" in the first place, to "the friction between the house and the hospital," and in the second to "the want of arrangements for the accommodation and the comfort of the nurses, notably in the matter of the food supplied." On this point it seems fair to say that there are many evidences that workhouse nursing is steadily diminishing in unpopularity, and that friction is becoming the exception rather than the rule, while even during the last five years there has been a great improvement both in the accommodation provided and in the food supplied by boards of guardians for Poor-law nurses.

### THE CHURCH AND NURSING.

We learn with interest that among the subjects to be brought forward at the Church Congress at Weymouth this year will be "Town and Village Nursing," and "The Visitation of the Sick." Now that the parish nurse is becoming more and more an institution—many of the clergy say that she is the most popular—the question of her qualifications is growing in importance, and we hope that in the discussion at the Church Congress stress will be laid upon this aspect of the matter. The clergy can do a good deal either to assist in raising or in lowering the standard of nursing, and we look to them to elevate it as far as possible by engaging for parish work fully-trained nurses. In the interests of the Church as well as of the sick under their care, they should endeavour to avoid employing an inferior class.

### GUARDIANS AND MIDWIVES' FEES.

The Islington guardians, replying to a communication from the London County Council, proposing payment of fees by the guardians in certain cases where a medical practitioner was summoned by a midwife to a poor person in difficulty or emergency, have expressed their readiness to deal with each case that may be submitted to them on its merits, and, if considered a proper one, to pay a reasonable fee. But they added in their letter the opinion that, in such a case a midwife should be instructed to send to the medical officer appointed by the guardians for the district in which the poor person resides, "as far as practicable." This is a sensible view of the matter, and will not prevent a poor woman from receiving medical attendance if one of the medical officers of the borough is not easily accessible.

### DEATH FROM SPOTTED FEVER.

The first victim among nurses of cerebro-spinal meningitis, popularly called spotted fever, was a monthly nurse at Berlin. She was engaged at her avocation at Rixdorf, a thickly-populated suburb of the German capital, when she was suddenly taken ill. The symptoms being of a mysterious character, the medical man ordered her removal to the municipal hospital. Unfortunately room could not be found for her in that institution, and the patient was despatched to the Charité Hospital, but she died on the way. Every precautionary measure was taken to prevent the spread of the disease, and each person who had come in contact with the victim was disinfected and will be closely observed.

### THE NEW MATRON OF PRETORIA ASYLUM.

We are officially informed that the Secretary of State for the Colonies has appointed Miss Catherine Borthwick to the important post of matron of Pretoria Asylum, South Africa. Miss Borthwick was trained at the Royal Hospital for Sick Children, Glasgow, the Royal Infirmary, Edinburgh, and for mental nursing at Stirling District Asylum, Larbert, N.B., where she has since been assistant matron.

### AN INSURANCE ORGAN ON THE PENSION FUND.

In an article on the Royal National Pension Fund for Nurses, the *Insurance Agents' News*, referring to the fact that the funds in hand at the close of the year were no less than £931,064, says that this "is a wonderful achievement for so young an institution." Our contemporary adds: "The advantages offered by the Fund are so great that we should like to see every nurse in the kingdom belong to it. For a very small yearly payment, well within the reach of every one who will make a little self-sacrifice, a nurse is enabled to provide for her declining years and for sickness, while, should she give up nursing to get married or to start in business, she can always withdraw her investment."

### ASSOCIATION OF ASYLUM WORKERS.

It is expected that the Archbishop of Canterbury will attend the annual meeting of the Asylum Workers' Association, which will be held on Friday, 19th prox., at the rooms of the Royal Medical and Chirurgical Society, 20 Hanover Square, W. The business will be of an interesting character, and will include the presentation of two gold and two silver medals, awarded by the Association for long and meritorious nursing service. Mr. Alexander Stephen, of Brislington House Asylum, Bristol, will advocate the institution of a mutual benefit fund for the asylum workers, and he intends to propose that all attendants on the insane in the United Kingdom be invited to become members, the fund to be independent of pensions that may be allowed from the asylums. At the instance of the Rev. C. W. A. Brooke, of Fulbourne Asylum, the committee of the Association will be requested to consider and report upon the advisability of amending its rules in reference to the status of associate members, the giving notice of motions at general meetings, and the possibility of giving greater interest in the Association to members living at a distance from London.

### BETHNAL GREEN NURSES' LEAGUE.

At the last quarterly meeting of the Bethnal Green Infirmary Nurses' League, the matron, Miss Dodds, received a large number of the members, including not only those who are at present on the staff of the infirmary, but also those who have left the institution and are engaged in nursing elsewhere. The proceedings included an address on the work of the Royal National Pension Fund for Nurses by Mr. Louis H. M. Dick, the secretary.

### CORNWALL COUNTY ASSOCIATION.

Not without reason, the Chairman at the annual meeting in Truro of the Cornwall County Nursing Association congratulated his audience on the satisfactory nature of the report. During last year the six new district associations formed in 1903 were firmly established, and two further organisations were started, namely, at Ludgram and St. Blazey and Par. The total number of districts affiliated to the association was 34, with eight Queen's nurses and 31 village nurses. The area covered by the operations of the association represents a population of 113,000, or about a third of the whole county. The Chairman stated that the association had trained 51 nurses at the comparatively small cost of £1,700, and he wondered whether £1,700 of public money had ever been more beneficently invested. Although the expenses for the year had been exceptionally heavy, the receipts had also greatly increased, the result of a special appeal for funds being that they had risen from £67 to £234. The total receipts were £1,740, and the total expenditure £652. Several speakers at the meeting expressed approval of the proposal to join in a movement for the provision of an emergency home for nurses with Devon and Somerset.

### SPANISH-AMERICAN WAR NURSES.

The annual meeting of the Spanish-American war nurses will be held in Washington, May 1 and 2. The special event will be the unveiling of the monument at the National Cemetery to the army nurses who died in 1898. The monument is in the form of a Maltese cross on the summit of a block of rough stone; above the inscription in the centre is a large wreath of bay leaves, part of which is resting on the inscription itself, the other part touching the cross, thus cleverly uniting the Cross and the Crown.

### THE UP-COUNTRY NURSING ASSOCIATION.

The report of the Up-Country Nursing Association which supplies nurses to the Europeans in up-country districts in India has just been issued. It was started in 1892, previous to which time, in many parts of the Punjab, Madras, and the United Provinces, it was impossible to obtain the services of a trained nurse. The staff now consists of 12 nurses—an apparently inadequate number, for several cases have had to be refused, some of them being cases of enteric fever. The nurses are engaged in England, the Association paying for their outfit, passage to India, and incidental expenses.

### THE SALISBURY NURSES' HOME.

At the annual meeting of the Salisbury Nurses' Home, the Mayor, who presided, said he was rather surprised to find that candidates for admission were not very plentiful. He saw from the report that it was possible, after a nurse had given her services to the Home for three or four years, to receive as much as £50 to £60 a year, in addition to board and lodging; and he thought that this should be a sufficient inducement for more young women to offer themselves for training. With regard to the financial position, the total income for the 12 months was £2,595, and after all payments had been made there remained a balance of £17. The payments included £85 bonuses to nurses. The staff at the present time consisted of 25 nurses and two probationers.

### THE POOR-LAW NURSING SERVICE AND REGISTRATION.

The King's Norton Guardians have passed a resolution, which, while generally approving of the Registration of Nurses, insists upon the necessity of safe-guarding the interests of the Poor-law Nursing Service. They accordingly recommend that the Poor-law Unions' Association should be invited to take action with a view to securing an amendment to the Nurses' Registration Bill providing that the Association shall have the right to nominate two or three experienced guardians, or clerks to guardians, as members of the General Council.

### PROGRESS AT WORTHING.

At the annual meeting of the Worthing Nursing Association, Lady Aubrey-Fletcher in the chair, several satisfactory announcements were made. Lady Aubrey-Fletcher, who caused some amusement by calling upon her husband to second a resolution the moment he entered the room, was able to show that the finances are in a sound condition. Last year's receipts included £36, the proceeds of a café chantant, and £20 from a concert organised by female Foresters. Reference was also made at the meeting to the fact that the Worthing Motor Omnibus Company had offered to afford facilities for the conveyance of the nurses. But the most encouraging intimation was to the effect that it may shortly be possible to furnish a house for the use of the Queen's nurses, and that already the nucleus of a fund for the purpose has been started.

### PROGRESS AT GLOUCESTER.

At the annual meeting of the Gloucester District Nursing Society the report was adopted. It contains some interesting features. The number of cases nursed during the year was 831, which necessitated 34,114 visits, an increase over the preceding year of 51 and 8,064 respectively. In addition 453 patients were attended by midwives, and 44 maternity cases nursed under doctors, the visits paid being 7,248. It was mentioned that twice a day trained nurses go their rounds to the sick, visits being paid to the rural parishes of the Union whenever required; and that the arrangements made with the school of cookery for the supply of dinners and nourishing food for the sick and convalescent at a small price are much appreciated. The advantages afforded to nurses by the Royal National Pension Fund were pointed out in detail in the report, and the chairman, in his address to the meeting, said that the District Nursing Society is loved among the poor and is beginning to be recognised by the rich.

### THE NURSE'S SHRUB.

A graceful act has been performed by the Crediton District Council. Recently an old nurse died in this town after a career of usefulness, in which she won the respect and confidence of all classes. She had in her garden a shrub bearing a small white flower, and it was her wish that, when she passed away, the shrub should be placed with the others in the enclosure adjoining the drinking fountain in the town. Respect has been paid to an honoured name by her wish being gratified.

### SHORT ITEMS.

Miss Jane Van Zandt, a graduate of the Margaret Fahnestock Training School for Nurses, Post-Graduate Hospital, New York, has been appointed head nurse of a new hospital in Beirut, Syria, connected with the Protestant Medical College.—Nurse H. L. Weaver, of the Trained Nurses' Institute, Weymouth, was presented at Easter with a silver medal for five years' service on the staff.

### The Nursing Outlook.

"From magnanimity, all fear above;
From nobler recompense, above applause,
Which owes to man's short outlook all its charm."

### THE QUESTION OF UNIFORM.

Not unnaturally, the self-respecting nurse resents the ease with which her uniform is imitated by those who have undesirable reasons for so doing. We may sympathise with her, but to assist her to protect the honour of the garb of her profession is not so easy. The simplicity which renders the best type of nurses' cloak and bonnet so suitable makes imitation easy, and admits of so little variety that even if the whole of the trained nurses and all those connected with recognised institutions were to adopt one particular style, and secure an Act of Parliament to register it, a deviation so slight as to be almost imperceptible would still enable the masquerader to wear the nurse's dress without much fear of penalty. But, whatever the difficulty of limiting the outdoor uniform for the trained nurse, we should still regret to see it abolished, as its use is attended with so many advantages, and the only possible distinction appears to be the official badge of each institution, which was suggested not long ago in our columns by a well-known matron.

In spite of the fact that the public no longer accept the wearing of uniform as a *sine qua non* of immaculate conduct, the wearer still retains the opportunity of carrying on work in districts where her presence might otherwise be unpleasantly questioned. Some of the hospitals are situated in not very desirable localities, but it is seldom that a nurse in uniform, quietly pursuing her way, meets with any disagreeable experience. This shows that nurses may themselves maintain the dignity of their uniform. This fact is, in our opinion, one of the most important arguments in favour of outdoor uniform. Not long ago a provincial matron wrote to us for our advice. She was in favour of the abolition of outdoor uniform for her own nurses, as she had had several unpleasant reports as to the flighty behaviour of her staff when off duty, and, owing to the uniform, they were identified with the institution to its discredit. We advised her without hesitation to insist upon uniform being worn out of doors at all times, but to educate the staff to respect their hospital and themselves, so that no discredit might attach to either. Nurses must remember that when they wear the uniform of an institution they are responsible for more than their own reputation. The necessity for such a feeling of responsibility is an advantage. It is part of the salutary discipline of training, and establishes a standard of dignity of conduct which is a valuable possession. A nurse should also remember that whilst her uniform secures for her both respect and consideration, it also renders her proportionately conspicuous. It gives her standing, but also demands of her a standard of conduct which may at times require her to exercise some self-restraint. Two nurses laughing and talking rather loudly in a public place would offend public taste and attract attention, whereas the same behaviour from two giddy girl members of the community might pass almost without notice or comment.

The world has its ideal pattern of conduct for a nurse, and if every individual member of the profession will remember this, the distinction between the sham and the real nurse will be so striking as not to depend upon the hall mark of a uniform. But, passing from the ethical advantages of the uniform, we turn to that practical one—economy. Almost, in some cases, in spite of herself, a nurse is a smartly and becomingly dressed member of society. Her dress, it is true, admits of a modification to suit the prevailing temperature, but beyond that it does not require or allow that variation which is the bane of those with little taste and less money. A nurse cannot, for instance, indulge in a wreath of red poppies on one side of her headdress, a group of pale pink roses nestling in her auburn hair, or a torn lace dress dragging in London's dirty streets in the morning. No one but the wearer of the unsuitable flimsy dress can defend it, but the admirers of the neat and business-like dress of the nurse are legion. Further, without the serviceable cloak, how many precious opportunities for healthful change and exercise would be lost? Think of hurriedly discarding the dress of the wards, and selecting and attiring in a costume suitable for the many various occasions to which you desire to devote that precious two hours off duty! In fact, the advantages of a nurse's uniform seem to us to be so convincing that little more need be said here. We have only one adverse criticism to make, and that is directed against the veil, which still forms part of some otherwise admirable uniforms. We cannot ourselves see its use, beauty, or economy. Our personal acquaintance with it in public vehicles has been somewhat baffling! And then one word to the wearers of uniform who lend to it much of its quality of charm or otherwise: Is there no way in which a nurse's locks can be disciplined so as not to emulate the wayward straying of the veil? We have heard comment in the streets more than once on the contrast between untidy hair and neat uniform. Nurses are intelligent and reasonable. They will take the little hint in the spirit in which it is intended, and remove the one blot which, it cannot be denied, too frequently mars an appearance to which otherwise no exception could be taken. A nurse must be of exceptionally untidy habits, to look otherwise than neat and businesslike in her uniform.

### The Nursing of Sick Children.

By James Burnet, M.A., M.B., M.R.C.P.Edin., Registrar, Royal Hospital for Sick Children; Clinical Tutor, Extramural Wards, Royal Infirmary; and Physician to the Marshall Street Dispensary, Edinburgh.

### V.—ON THE MANAGEMENT OF RESPIRATORY CASES.—(*Continued from p.* 37.)

In the last lecture we considered some general points in connection with the nursing of respiratory cases. We have now to look for a little at some methods used in the treatment of such cases, and to indicate as clearly as possible how these should be practically carried out.

### How to Give an Inhalation.

Inhalations are specially useful for children who are suffering from inflammation of the upper respiratory passages—namely, the larynx and trachea. At the same time they do good in certain cases of bronchitis, while they are of undoubted value in children affected with tuberculous disease of the lungs. In diphtheria inhalations are sometimes ordered to be given, and in cases of measles or of scarlet fever they may be prescribed. The mode of giving an inhalation is very simple and easily carried out. The child should be wrapped up in blankets, and either kept in bed, or preferably seated on the nurse's knees. About a pint of boiling water is poured into a somewhat narrow jug, and over the top is placed a towel, twisted into the shape of a cone and then pinned. The end of this cone is brought up to the child's nose, and he is made to inhale the steam. By adding a few drops of eucalyptus oil or of creosote to the water an antiseptic inhalant is obtained which is of very great value in many cases. The child often resents the treatment at first, but a little persuasion and patience on the part of the nurse will usually overcome any resistance.

### Fomentations.

A fomentation is really a modified form of poultice, and consists of a piece of flannel wrung out of hot water and applied to the affected part. Fomentations are sometimes termed "stupes." In the case of children fomentations are most frequently used for throat affections, though some physicians are in the habit of using them in cases of pleurisy and of pneumonia to ease the pain. A fomentation cloth is made by taking a large piece of fairly thick flannel and folding it once. This is then placed in a basin of boiling water and allowed to remain there till it is thoroughly saturated. It is then removed by means of a piece of wood, placed in an ordinary towel, and thoroughly wrung out by screwing it round. Care should be taken that the fomentation is neither too hot nor too wet. It should be tested by placing it against the cheek before applying it to the patient. When in position it should be covered over with a piece of pink jaconet, over which a sheet of cotton wool is placed. As soon as the fomentation begins to get cold a fresh one must be applied. When their application has been interrupted the skin should be gently dried, and it is well to apply some olive oil, and then to cover up the part with a layer of cotton wool. The essential point to attend to in applying a fomentation is to avoid scalding the child.

Fomentations may be medicated by adding a dessert-spoonful of turpentine to a pint of boiling water and placing the flannel in this. Never sprinkle turpentine on the fomentation cloth just before applying it, otherwise the child's skin will be severely blistered. In the case of other substances, such as lead and opium or belladonna, the physician always indicates the quantity to be employed, and the nurse sprinkles it over the fomentation cloth after it has been wrung out of the boiling water.

### Poultices.

Poultices are not so frequently ordered now in the case of children, as, unless the nurse is very careful in their application, they are apt to cause very extensive burns. Poultices may be used for the relief of pain, and for the arrest of inflammatory processes. Accordingly they are of special value in respiratory diseases.

For a poultice we require a piece of old linen, a sheet of pink jaconet and some cotton-wool. The most common form of poultice employed in chest cases is the linseed and mustard one. This is prepared as follows:—A quantity of boiling water is poured into a bowl and then sufficient linseed-meal is added until we have a fairly thick paste formed. This should be perfectly smooth and of a uniform consistence throughout. The required amount of mustard is then made into a thin paste with cold water, and either spread on the top of the linseed or preferably mixed up into the hot mass. The whole is then spread evenly upon the piece of linen, and care should be taken that the poultice is not too heavy. Its thickness should certainly never exceed a quarter of an inch. When ready it is rolled up in flannel and taken to the sickroom. It is first tested by placing it against the nurse's cheek to see that it is not too hot; and it is well to remember that after a few minutes the presence of the mustard tends to make itself felt even more than at first. After being applied to the patient, the poultice is covered over with jaconet, and on the top of this a sheet of cotton-wool is placed. The whole may be kept in position by means of a broad domette bandage. A poultice should never be kept in position for more than from twenty minutes to half-an-hour; and when it has been removed the patient's skin should be wiped dry and a little olive oil applied. A sheet of cotton-wool is then laid over the part.

### The Steam Tent and how to Extemporise it.

This apparatus is chiefly used in cases of laryngitis and of severe bronchial affections. To make a tent we may put a draught screen round the upper end of the crib, or a clothes screen will serve the same purpose; over the top an ordinary sheet is placed. It is best to use a bronchitis kettle possessing a long spout, but a very simple form of apparatus is made by the Sanitas Company, and this can be readily obtained from any chemist. Failing these, however, an ordinary kettle will do quite well. Any tinsmith will make a long spout to fit on to this, or for temporary use a long roll of thick brown paper will prove quite effectual. The top of the long spout must in any case be brought up into the bed so that the steam may play round about the patient. The steam may, if thought advisable by the medical attendant, be made antiseptic by adding some sanitas oil or eucalyptus oil to the water in the kettle. Care must be taken not to scald the patient, a misfortune which may readily happen if the steam is brought too near the face. If a lamp is used for boiling the kettle the nurse must see that this is not upset, as serious accidents sometimes happen in this way. Lastly, it is well to point out that the patient is apt to be chilled if the steam is suddenly taken away, and so if it has been used for some time it is well to intermit it, if possible, during the day time, rather than to remove it at night, when the atmospheric temperature tends to be lowered.

### The Aspirator.

This instrument is employed for the removal of fluid from the chest in cases of pleurisy and of empyema. It consists essentially of three parts: a bottle with cork, an air-exhausting pump, and a trocar and canula attached to the bottle by means of an indiarubber tube. The nurse has certain duties to perform when the operation of aspiration is about to be performed. She must see that the skin over the part where the trocar is to be introduced is rendered thoroughly aseptic. Then she must have ready for the physician's use some ether for application to the skin, or, better still, a chloride of ethyl spray. She must have the aspirating bottle thoroughly washed out with carbolic lotion (1 in 20) and finally rinsed in sterilised water. She should have the air in it exhausted by means of the pump, and, if asked by the physician to do so, she may even fix up the trocar and canula ready for use.

When the physician is introducing the trocar the nurse must open the stop-cock (c) to allow the trocar to pass through the canula; and when the trocar is withdrawn she must be ready to shut the stop-cock immediately, otherwise air will enter the chest. She has, in addition, to make sure that the stop-cock at A is closed and that the one at B is also shut off before the physician introduces the trocar. When the canula has been inserted between the ribs the physician opens the stop-cock at B and this allows the fluid to be aspirated into the bottle. When the operation is ended, the nurse is ready with wool and collodion to close over the opening in the chest-wall at the moment when the canula is withdrawn by the physician.

### The Nurses' Clinic.

### DISPENSING: MAKING OINTMENTS. BY A CERTIFICATED DISPENSER.

The making of ointments is in itself generally a very simple matter, but one requiring, nevertheless, some care, and a certain amount of time and patience. Ointments are made up of two parts—first, the medicinal substance consisting of one or more ingredients; and, secondly, the basis, which is composed of lard, paraffin, lanoline, or wax and oil. There are 44 official ointments, 21 of which contain lard in some form. Ten are mercurial ointments. Vaseline is official under the name paraffinum molle. When vaseline is prescribed the yellow should be used, never the white, unless specially ordered. Lanoline is also official, as adeps lanæ hydrosus. "Cold cream" comes under the name of "unguentum aquæ rosæ" (rose-water ointment), and is made up of white beeswax and spermaceti, almond oil, rose-water, and oil of roses.

The best way to test an ointment to see that all has been thoroughly incorporated with the basis, is to spread a small portion of it on to a piece of thin paper and hold it up to the light. If small specks show, it proves that it has not been thoroughly mixed, and that more rubbing up will be necessary; if, on the other hand, after two or three portions have been tested, all is quite clear and smooth, it shows that it is perfectly done. Great care must be taken in getting it quite free from grit, as it may scratch a wound to which it is being applied and do more harm than good. Special care must always be taken with eye ointments.

In weighing out ingredients, first weigh the medicinal substance on a piece of paper on the scales and put it on the slab or mortar where it is to be made up, then measure the basis on another piece of paper of equal size to keep the balance correct; this prevents the basis from greasing the scales, and it also lessens the risk, in the case of a poison, as hydrarg. perchlor., or of a strongly-smelling powder, as iodoform, of any sticking to the scale pan. After having weighed out all that is required, the next thing to do, in the case of a powder, crystal, or extract, is to grind and rub it up well in the mortar, then add the basis little by little, until the whole is thoroughly incorporated, scraping round the mortar every now and then with a long spatula that none may be wasted by being left sticking to the sides. It is better to make a rule of always using a bone spatula, or a boxwood knife, rather than a steel one, as some substances, such as all mercurial preparations, are ruined by the touch of iron. It is a general rule to make up large quantities of ointment in a mortar, but it will be found easier, when only a very little is required, to do it on a porcelain slab with a spatula after first grinding the powder or crystal in a small mortar. In mixing two ointments, or an ointment with a liquid or oil, the simplest way is to mix them on a slab. When using a mortar, care should be taken to have one large enough to hold a great deal more than is required, otherwise a great mess can hardly be avoided. A good dispenser leaves all the vessels and implements as clean as possible after using them. An untidy dispenser is a bad dispenser. Extracts should generally be rubbed up on a slab. Watery extracts should be rubbed smooth with a little water (as little as possible) and spirituous ones with a little diluted spirit. Resinous extracts can also be thinned with spirit, or finely

**THE NURSES' CLINIC**—*Continued.*

powdered, put into melted lard, and stirred until cold. If an extract is too hard to work in easily the mortar should be well warmed with hot water, quickly dried with a cloth, and it can then be rubbed smooth before the basis is added.

In the case of powders and crystals, sometimes something will be required to facilitate grinding before adding the basis—very insoluble salts as zinci sulph. or nitrate of silver are best rubbed down with a little oil. Very soluble or deliquescent salts, as pot. iod., pot. carb., or zinci chlor., require a little water, if this does not hurt the other ingredients. Antim. tart. should be used dry. Carbolic acid should not be used in a hot mixture, as it crystallises out on cooling. It should be made cold by mixing the liquefied acid (acid carbol. liquefactum) with the basis. Hydrarg. perchlor. is best rubbed up with two minims of glycerine to each grain of hydrarg. perchlor. before adding the basis. If ordered with potassium iodide, rub both powders together until quite smooth and then add the basis, as potassium iodide increases the solubility of mercury. Iodine should have a few drops of spirit added after having been rubbed up with its own bulk of the basis. Iodoform should be dissolved in melted fat at a low temperature. Resorcin must be rubbed up separately with some of the basis, and the other ingredients added afterwards, or it is apt to colour the ointment. Thymol should only be combined in a state of solution. Veratrine, unless carefully rubbed up with a little olive oil, raises a dust which is very irritating to the nostrils. Alkaloids, as aconitine and atropine, are best dissolved in a very little spirit, then gradually stirred, in a mortar, into the fat. Ointments containing oleates should not be mixed in a metal mortar or with a steel spatula, or chemical action will occur. Volatile liquids should be added after the other ingredients are well mixed in order to lose as little as possible by evaporation. Tinctures are not easily combined with fat; ordinary soft lard will take up one-fifth, hard lard will take up one-sixth of its weight of tincture. A little soap powder, if permissible, greatly facilitates the combination.

Sometimes the ingredients require melting, as wax or hard paraffin; in this case the hardest substance should be melted first, the other fats or oils next added, and the mixture stirred till cool; if it has not been overheated it may cool without stirring, but if it contains something which may separate on cooling it should be poured into a warm mortar and triturated until it becomes pasty. Ung. zinci should be carefully stirred, otherwise the zinc falls to the bottom and when cold the top would be pure fat, while at the bottom of the mortar the ointment would be far too strong. Liquids are often added to impart a cooling property to ointments. When a medicinal substance is ordered to be made into an ointment, and no particular basis is specified, the general rule is to use a white one for white substances, and yellow for coloured ones, but this rule does not apply in the case of eye ointments, as in the following:—

Atropinæ gr. i.

Vaseline flav. (yellow) ʒij.

M. Ft. ung. pro oculo.

White vaseline is said to be irritating to the eyes.

When finished, ointments are generally sent out in round covered porcelain pots with a piece of waxed paper between the ointment and the lid. If, however, an ointment is of too liquid a consistence, it should be put into a wide-mouthed stoppered bottle. In hospitals and dispensaries common willow boxes are usually employed.

### Preparing a Patient for Operation.

### EXAMINATION QUESTIONS FOR NURSES.

The question was as follows:—"If permitted by the doctor to choose your own means and hours, in what way would you prepare a patient for operation with regard to clearing the bowels?"

### First Prize.

In order to prepare a patient effectually for operation it is advisable to begin, if possible, two days before.

Supposing the operation is to be performed on Wednesday at 10 a.m., the patient should be given castor oil between 5 and 7 p.m. (according to the time of his evening meal—either several hours before or soon after) on Monday—1 ounce for a man, from 4 to 6 drams for a woman, from 2 to 3 drams for a child. Given in the juice of half a lemon and swallowed whole, there is little or no taste.

If the patient is susceptible to aperients this will probably act before 11 p.m., or if he is not, it usually acts early in the morning; this generally enables him to obtain a good night's rest.

About 8 p.m. on Tuesday give an enema of soft soap and water, from 1 to 2 pints, according to the amount the patient can retain. This is best given with a Higginson's enema-syringe to which a soft rubber nozzle has been attached. If the operation is not to be performed until the afternoon, give the enema about 7 or 8 a.m. on the same day.

In the case of much previous constipation, or operations on the rectum, it is advisable to give a second enema. Thus, for the morning operation give one the middle of the previous day, followed by another about 8 p.m. For an afternoon operation, give one the previous evening in addition to the early morning.

If, as sometimes happens in hospital practice, one is unable to commence preparations until the day before operation, give the dose of castor oil immediately upon the admission of the patient, followed by an enema early in the morning, at least four hours before operation, and if possible longer.

In any case, avoid as much as possible interference with sleep—a good night's rest is of the utmost importance before an operation.

This is a plan which I have followed for several years, and have invariably found it entirely successful. "Maria."

### Second Prize.

Supposing I am preparing a patient for afternoon operation I always give castor oil, ℥ i at 4 a.m. on the preceding day; if this does not cause the bowels to act well (which it nearly always does) I repeat the dose at 3 p.m. I give a simple enema the next morning. As a rule the bowels act well during the day on which oil is given, and the patient has an undisturbed night before operation when the rest is badly needed.

"Nance."

### The Prize-Winners.

"Maria" and "Nance" are successful as sending the best all-round answers; "Maria" mentions the advisability of beginning treatment 48 hours before operation when possible, but also states what she should do for the best when only informed of the impending operation the day before.

### Honourable Mentions.

A large number of good answers have been sent in and five have been selected for honourable mention—"Belinda,"

"Central," "Practical," "Kathleen," and "Lanoitan." "Belinda's" paper is extremely good and practical, but I think calomel except in rare cases is not advisable.

Faults in the Papers.

As usual several nurses write without appending name and address. Several others err in reading the question so carelessly that they imagine it means that they themselves would be allowed to fix the hour of the operation—a degree of amiable complaisance hardly to be expected from a busy surgeon.

Question for April.

In a case of fractured thigh put up with a long splint and extension, and in a case of hip disease treated with splint and extension, what precautions should you take for the prevention of splint sores? The Examiner.

Rules.

The competition is open to all. Answers must not exceed 500 words, and must be written on one side of the paper only, without divisions, head lines, or marginal notes. The pseudonym, as well as the proper name and address, must be written on the *same* paper, and *not on a separate sheet*. Papers may be sent in for 15 days only from the day of the publication of the question. All illustrations strictly prohibited. Failure to comply with these rules will disqualify the candidate for competition. Prizes will be awarded for the best two answers. Papers to be sent to "The Editor," with "Examination" written on the left-hand corner of the envelope.

In addition to two prizes honourable mention cards will be awarded to those who have sent in exceptionally good papers.'

N.B.—The decision of the Examiner is final, and no correspondence on the subject can be entertained.

Any competitor having gained three prizes within the current year shall be disqualified from taking another until 12 months shall have expired since the first prize was gained.

### M. Mesurier and the Nursing Question in Paris.

The Assistance Publique de Paris is divided into three departments—hospitals, infirmaries, and private or parish relief. There is a council which directs and rules the income and administration of these establishments. M. Mesurier is at the head of this council, he is the king of the municipal hospitals and infirmaries of Paris, his title is Directeur Général de l'Assistance Publique de Paris, late Minister of Commerce.

The position he now occupies has brought him to the front, and he is said to have done in two years what others have not been able to accomplish in ten. Under his control the hospitals are improving and the nursing profession is rising and becoming more respected in Paris. It will be remembered that some twenty years ago Paris secularised its hospitals. It was a political and not an administrative or professional stroke, and was anti-clerical pure and simple.

At first the nuns were regretted by the doctors, the patients, and the public—and no wonder, for the nursing staff was filled by the lowest class of women. Latterly this has changed; a preliminary examination is now compulsory before a candidate is accepted, and professional examinations have to be passed before the candidate is acknowledged as a pupil-nurse. Those who pass the highest examinations (and they are both theoretical and practical) obtain by right the higher posts. Thus M. Mesurier is raising the standard of nursing and holding out inducements to the better-educated women. He has also revised the rules for the nurses and *surveillantes* (heads of wards) which had become obsolete or simply traditional, and has sent them around in printed pamphlet form.

"The duties of the *surveillantes*," he writes, "do not finish when they have seen to the material wants of the sick. They have also a moral mission to accomplish at their bedsides. They must say the comforting words which their hearts dictate. Towards the aged and infirm they must be armed with that patience which enables them to give a sympathetic ear to their repeated and unceasing complaints. . . . It is particularly at the departure of the patient that the *surveillante* must be most awake to her duties. It is on her that devolves the duty of reminding the patient of the dietary he must follow and all the precautions he must take during convalescence to guard against the possibility of a relapse. It is for her to point out to the director the sick who require immediate help, or those who have to be driven home and those who need a nurse to accompany them." . . . With regard to liberty of conscience and religious convictions of the patients M. Mesurier says: "It is the duty of the administration to assure to the sick complete liberty of conscience and quietness of mind. The ministers of the different religions and denominations can only enter an establishment through a call from the director. They must not address themselves to any patients except those who have expressed a special desire to see them. They are to baptize no children without the express wish of their mothers. The lady visitors who have received special permission to visit the sick must not overstep their boundary, which is to bring moral comfort quite free of all political and religious questions. It is for the *surveillantes* to inform the director in case any of the lady visitors infringe these rules and interfere in any way in matters which do not concern them.

M. Mesurier frequently visits the hospitals and tries to judge for himself of complaints and matters which have been brought before him. In fact, it was he who sent M. Montreuil, the director of the Salpêtrière Hospital, to study the nursing question and hospital administration in the London hospitals, and on his return, having given a glowing and enthusiastic account of what he saw, it was M. Mesurier who organised a commission to study the question still further. Lately the widow of Emile Zola, the great novelist, went to him in order to ask his advice as to disposing of a beautiful property she still possessed, but felt unable to keep up. Her wish was to present it to the Government for the use of aged and needy literary men. She was very much astonished when M. Mesurier asked her whether she would not reconsider the question and give it for the use of worn-out and convalescent *infirmières*. "I came away enchanted and full of joy," she relates, "at the thought that our beloved *Médan* was going to be given to those women who are devoting their lives to the care of the sick, and I felt that of all things it was the one my dear husband would have liked best."

Madame Zola's gift is the very first act of kindness shown by the public to the servants of the sick in Paris. Let us therefore hope that other benevolent people will follow her example, and by a little kindness and forethought make those overworked, underfed, ill-lodged, much-maligned women feel that the outside world is beginning to recognise their hardships and sufferings, and make them thus feel the dignity of their profession, whereas now, so far, the very last thing a woman chooses as a means of livelihood is to become an *infirmière*.

M. Mesurier is now building a college for nurses in that vast enclosure of the Salpêtrière. What its practical results will be still remains to be seen, but it will unquestionably have the result of raising and dignifying the profession, as only educated women will be admitted as candidates, thus putting nursing on an intellectual and educational basis.

The nursing books which the municipal nurses are obliged to study consist of five volumes containing eight subjects, the main point being that there is a uniformity of curriculum and standard. Unfortunately, the books are too difficult for the present average nurse, and there are too many subjects crowded into the first year; but now that the question has been taken up, doubtless in the course of time things will evolve and correct themselves.

### The New Mission Hospital in Corea.

BY A BART.'S NURSE.

My husband and I arrived in Chemulpo exactly a year ago, to start our work as medical officer and lady superintendent to St. Luke's Hospital, belonging to the English Church Mission. We had considerable difficulty in getting here owing to the Russo-Japanese War having just broken out, but after some delay in Shanghai and Nagasaki, we reached our destination six weeks after the battle of Chemulpo, and were a little surprised to find everything entirely peaceful. Most of the foreign residents were flying flags over their houses, the masts and funnels of the *Variag* were to be seen in the harbour, and in the streets a large number of Japanese soldiers, but these were most orderly and quiet.

### Three Nurses to One Patient.

Our little hospital, which had been previously closed for some time, had been lent to the Japanese for the use of the Russian sailors wounded in the battle. These sailors were nursed by members of the Japanese Red Cross Society, to which Society nearly all the Japanese ladies resident in Chemulpo belong. So many willing helpers were there that, in a photograph taken when all the patients were convalescent, there are about three nurses to each patient. Not only did these ladies render valuable assistance in nursing, but they provided warm comforts and soup for the men during that very cold season. These, however, had all gone before we arrived, and, indeed, during their stay they were in imminent danger of the hospital falling in on the top of them, for the foundations had given way in some places, and Chinese workmen had already begun to pull down some part of the building. Since then the hospital has been almost entirely rebuilt, during which time our work was confined to out-patients, who came in considerable numbers from the first and were treated by means of an interpreter.

### European Style.

Now, we have doctors' quarters, nurses' quarters, two large male wards, two private rooms, waiting-rooms, surgery, dispensary, operating theatre, sterilising-room, laboratory, kitchen, bath-room, nurses' sitting-room, etc., all conveniently built in European style, well lighted and ventilated, and only waiting to be put to their fullest use till we have at least one trained nurse and a dispenser, which we hope will be at no very distant date. Our operating theatre is built with two small rooms opening into it—one for sterilising, which we always do immediately before our major operations, and the other for the surgeon to wash his hands in. We have a modern asceptic operation-table and dressing-wagon, which were made in America and presented to our hospital in Seoul by an American gentleman, and have been handed down to us. Out-patients are only seen between 9 and 12, except urgent cases, and at present we have only one ward open, as so much of our time must be given to the mastery of this most difficult language, without which it is impossible to do any really satisfactory work.

### The Corean Doctors' Cure.

We get a great variety of cases, almost as great a variety as may be seen in any ordinary out-patient department, only that by far the commonest complaint is worms, from which every Corean seems to suffer more or less. Santonin has to be given always on the premises, as it can be sold for a considerable sum, and is in great request. We have a good many minor surgery operations, besides the more important ones, which have to be taken in; abscesses of all kinds are very common, being frequently the result of a Corean doctor's cure for all pain—the needle—in which Coreans have great faith.

### Boys as Nurses.

For assistants we have two Corean boys who worked for several years in the Mission hospital in Seoul, which is now closed for lack of men and funds. One of these boys knows a little English, about as much as we know Corean, and through him we extract histories and symptoms more or less satisfactorily, and give directions for treatment. These two boys are our nurses, dressers, assistant dispensers, and, in fact, everything; and, considering the slovenly habits of the Corean in his native state, they do exceedingly well. They go round with the doctor, prepare for and assist at operations, do practically all the dressings, make ointments, powders, and pills, and one or other of them is on duty in the ward day and night. I give all the anæsthetics, help with the female patients and superintend the linen and stores of the hospital, but we feel the need of a nurse very much, to be on the spot more than I can be, and to see that the boys do their work and carry out the directions given them.

### No Female Ward.

At present we have no accommodation for female in-patients in our hospital, but I hope that some day we shall have a small female ward. It is impossible in this country to have male and female wards under one roof, unless they can be so entirely separated that the patients will never meet, and our building does not allow of that. We have one small room in a Corean house in the compound into which we can put an urgent female case, and our Bible-woman, who lives in the house, looks after it, but so far we have had very little use even for that one room. Respectable women in Corea seldom, if ever, go out, and, unless they are old, they are not supposed to see any man save their father, husband, and brothers. In spite of this fact they do come in very fair numbers for treatment, both for themselves and their children; though, if they cannot walk they have seldom any other means of coming, and are not considered of sufficient importance to be brought. Their lives are spent in washing and getting up the beautiful white garments which a Corean gentleman invariably wears, and it is almost impossible for a woman to leave her home unless she is a widow.

### To Nurses.

We invite contributions from any of our readers, and shall be glad to pay for "Notes on News from the Nursing World," or for articles describing nursing experiences at home or abroad dealing with any nursing question from an original point of view according to length. The minimum payment is 5s. Contributions on topical subjects are specially welcome. Notices of appointments, letters, entertainments, presentations, and deaths are not paid for, but we are always glad to receive them. All rejected manuscripts are returned in due course, and all payments for manuscripts used are made as early as possible after the beginning of each quarter.

### Wants and Workers.

Will some kind friend, having a disused dressing-gown and old slippers, allow District Nurse Haveringland, Norwich, to have them for a poor helpless patient suffering with dropsy, who cannot be dressed and is too poor to purchase?

### A Nurse's Experience of the Earthquake in India.

Early on Tuesday morning, April 4th, at 6.10 a.m., writes a staff nurse at the European Cottage Hospital, Mussoorie, we were awakened by the sensation that we were being tossed up and down in our beds, the windows began to rattle, the water in our baths was splashing all about, and we felt for all the world as though we were in a railway carriage and going down hill at a good pace. I, who had never experienced a similar sensation, thought that it must be a landslip, and that before we could get out of the house we should be precipitated down the "khud"—or the side of the mountain—but then others who knew what it was to feel an earthquake shock told me what was happening. It lasted nearly three minutes in all. The Cottage Hospital had two large cracks in it, one from the roof to the foundation, and the other slighter. Close by a house fell in, crushing five native servants who were asleep, and they were all killed; a native postman was so injured by a skylight falling on him that he died the same night, and some soldiers in Landour—the military part of Mussoorie—were injured and had to be taken into hospital. One of the most fashionable of the hotels is so much injured that it is uninhabitable; likewise the convent and the new Roman Catholic church (which it was hoped would be finished shortly) are both much damaged. There are several houses which will have to be almost rebuilt, and every one is afraid that it will be a great loss to this favourite hill station, as most likely people will be afraid to come here. There were several small shocks during the rest of the day, about eight in all, but they only lasted a few seconds. Most of us went to bed partially dressed that night, but there was only one small shock felt at midnight, and I am glad to say that it was the last, and we all sincerely hope that we shall have no more of it. Certainly such an experience is sufficient for me to have once in a lifetime. The earthquake was preceded a few days before by two severe thunderstorms, with very vivid lightning.

### The Nurses' Bookshelf.

Midwifery for Nurses.

Messrs. J. and A. Churchill will publish at the end of this month the second edition of "A Short Practice of Midwifery for Nurses," by Dr. Henry Jellett. The book has been considerably amplified as regards both text and illustrations, the latter amounting now to 134. New features introduced are four coloured plates, and a glossary of terms employed in the work.

Notes for Maternity Nurses. (London: Allen and Hanbury. Pp. 80. Price 1s.)

Messrs. Allen and Hanbury have brought out a neat little volume, in pocket-book form, containing information as to management of labour, many useful recipes for the sick-room, and hints on the feeding of infants. Space is provided for engagements, addresses, etc. The size is convenient, and the book ought to be popular with maternity nurses.

Our Baby: For Mothers and Nurses. By Mrs. J. Langton Hewer. Ninth Edition. (Bristol: John Wright and Co. Pp. 158. Price 1s. 6d.)

Mrs. Hewer's little book has been so long and so favourably known to the public as scarcely to require additional recommendation. This edition, however, has been thoroughly recast so as to still further increase its usefulness. As a concise and handy guide to the feeding, clothing, and training of infants in a state of health it is both reliable and complete, while the hints on their ailments and their treatment are characterised by moderation and common sense. The directions are always clearly expressed and its moderate price should ensure it a wid circulation.

### Practical Hints.

We welcome notes on practical points from nurses.

### A CHEAP NIGHT LIGHT.

District nurses might find of use a cheap night light which can be made in the following manner:—Fill a small tin box with salt; in the lid of the box bore a small hole; place on the top of the salt a piece of cotton-wool soaked in paraffin, and draw a small portion of the wool through the hole in the lid. This makes a very satisfactory night light. The wool requires resoaking sometimes, but otherwise it lasts as a burner for some time.

### LIFTING HANDLE OR BAR FOR PATIENTS IN BED.

At the recommendation of the medical adviser I had one of the usual pattern of cross handle and cord made for my patient, but found it of little practical service, although better than nothing. After some trials, I came to the conclusion that a handle of any kind was a mistake, the fixed height making it out of reach in some positions, in others it was was either too high or too low to be used without strain. After several trials I finally adopted an ordinary clothes line of Italian hemp, with a row of double knots, *i.e.*, one outside the other, so as to form a larger knob for the hand to grasp, these being about 2½ inches clear apart, so as to allow the hand to rest on two at once, and divide the strain. The knots should be about twelve in number, the highest at the highest point which can be reached, the lowest at the level of the bed clothes, the free end being long enough to hang over the bedside, or to loop on the bed head, or on a wall if the bed be against one. This can be firmly and easily grasped by either hand, or by both, in any position, at the best height for power without strain, and is especially advantageous for turning from side to side, or altering the position. The double knot is necessary, a single one is not large enough. The common clothes line is too rough to be comfortable; but the Italian hemp is almost as smooth as cotton cord, does not show marks, and is easily obtained. The best hook is what is known as a "bacon hook," such as is used for hanging joints or hams, and this, of course, must be screwed into a rafter or beam in the ceiling, the position of which is easily found by the dead sound when the ceiling is tapped with a hammer. But if doubtful it is safer for a nurse to employ a carpenter for this. When properly screwed in it will carry the full weight of two heavy persons hanging on the rope.

### Presentations.

Bradford Children's Hospital.—Miss Flora B. J. Cameron, who has been matron of the Bradford Children's Hospital for four years, having been appointed matron of the Manchester Children's Hospital at Pendlebury, has been the recipient of several handsome presents, including a silver afternoon tea service from the members of the Board of Management, a silver travelling clock from the Ladies' Committee, a silver flower vase from the nursing staff, and a silver photograph frame and silver and tortoise-shell paper-knife from the domestic staff.

Bridgend Cottage Hospital.—At the quarterly meeting of the Committee of Bridgend Cottage Hospital this month, the chairman, Colonel Turbervill, on behalf of the committee, presented the retiring matron, Miss Tanner, with a silver Queen Anne teapot on a handsome silver stand, both suitably inscribed, as a memento of her three years' work, on the occasion of her marriage Miss Tanner was also the recipient of a pearl and turquoise bracelet from the medical staff, and of numerous other gifts from individual members of the committee.

Lethbridge Sick Nursing Society.—Nurse Harrod, who was for three years associated with the Lethbridge Sick Nursing Society, and has lately resigned, has been presented by the subscribers and committee with a handsome gold watch, towards which no fewer than 796 inhabitants of Sheerness contributed. The initiative was taken by representatives of men inside and outside the dockyard, the highest subscription being 10s., while there were many shillings and a great number of pennies.

### Bird Life near London.

### A SATURDAY'S RAMBLE.

Whether free to visit the country or not, there are few residents in town who cannot enter into the delights of the country. The harder a man or woman works in town the greater to them is the attraction of the country; usually, indeed, there is no form of relaxation so wholly beneficial to an overworked and tired urban, as a country ramble, provided it can be made to afford some object of individual interest. To the naturalist, of course, such objects are legion. At the present season when spring is asserting itself everywhere, the beauties of the foliage are increasing daily and the activities of animal life are full of pleasant instruction to those who have eyes to see and minds to think and observe closely.

An account of such a ramble on Easter Saturday may then interest the many who are chained to town as well as those who love the country.

Taking train to Woking and walking about three miles one comes to a common which stretches for miles and is very beautiful. It is marshy in places and full of bird-life of all descriptions. We soon became aware of the presence of that most beautiful of English birds, the green woodpecker, and its nest deep down in the opening in an oak tree which it had made was not far to seek. We noticed on crossing through a hedge that a grey plover was standing some hundred yards off, and soon indicated by its antics that it was anxious to attract attention to itself. This made us feel certain that its nest could not be far off, and so we kept perfectly still, observing our handsome friend on the ground ahead. In a few minutes the plover rose, then later its mate rose too, some 200 yards away. They circled round and as we began to search the rough, marshy ground they became more and more demonstrative. In about half an hour we found the nest placed upon a tuft of rough grass, and it contained two chicks and an egg in course of hatching. The chicks were beautiful little things covered with brown, hairy feathers, and most attractive to look at. A couple of hours later we found the third chick hatched out, but not a trace of the shell. It is the habit of the old birds to get the chicks out of the nest at once, and we found one had already gone, but we discovered it in the grass and replaced it. They would all be off by the next morning. It is strange to think of a young thing which faces the world so readily and strongly three hours after birth.

This incident shows that instinct may become so strong in birds when they are anxious for their young that it amounts to a system of tactics carefully planned and put in force for their protection. What had happened was this: The bird we first saw was the male doing "sentry-go" whilst the female was sitting. We entered the marshy ground about 20 yards from the nest. The sentry did his best to get us to approach him, and so draw us away from the place where his mate was sitting. When we remained stationary, knowing the habits of the birds, he called to his mate, when she quietly left the nest, crept along the ground for many yards, passed her mate, and did not rise until he was attracting attention in the air. A friend, who was with us, was entirely deceived, and insisted that the nest must be near the place where we first saw the sentinel standing. His prolonged search proved fruitless, as we explained to him would be the case, and the nest was found within a short distance of the place at which we first entered the marsh. Instinct, which compels reasoned action, such as we have described, is very remarkable and instructive. Fired by this incident we kept a sharp look-out for snipe, which we were told were to be seen in this district. We saw none, but towards sundown we noticed a bird flying near the ground which from its zig-zag flight we took to be a snipe. On making careful search, in about half an hour we found a snipe's nest with four eggs. It is not usual, we believe, to find snipe breeding so far south, and if this is not the first recorded instance of the kind, it is at least remarkable to have met with it within 25 miles of London. All lovers of birds will appreciate the happiness and interest which accrued to us from this afternoon spent in observing these common objects of the country. The pleasures of life can hardly be better secured than by such a Saturday's ramble on a Surrey common.

### Everybody's Opinion.

[Correspondence on all subjects is invited, but we cannot in any way be responsible for the opinions expressed by our correspondents. No communication can be entertained if the name and address of the correspondent are not given as a guarantee of good faith, but not necessarily for publication All correspondents should write on one side of the paper only.

### THE UNTRAINED MATRON AT WORK.

"An Onlooker" writes: In reading your notes on "The Untrained Matron at Work" in your widely-read journal, I cannot help feeling that several remarks (however well intentioned) may lead some readers to come to an altogether wrong conclusion, both as regards the treatment of the case in question and also the efficiency of the matron in charge of the infirmary at St. Albans. Upon closer investigation I think you will find that the patient to whom brandy was administered was not, at the actual time of his admission, under the influence of alcohol, and entire approval of the treatment adopted has since been expressed by medical men. The matron of this institution is known by many to be not only most humane and conscientious, but perfectly capable of fulfilling all duties connected with the sick under her charge. She may not be "trained" in the accepted sense of the term, but I venture to urge that years of experience, coupled with continuous service, should be estimated at their true value. Having held her present position for the past 13 years, the matron was prior to her appointment at St. Albans, for a similar number of years actively engaged in all branches of nursing, including both mental and accident cases. I will not enter upon the question of what may or may not be a case requiring the immediate attention of a doctor. Much may be said on both sides, but I may point out that the fact of a person's being fully trained, or even a medical man, may not always avert the disaster of an error in judgment. Knowing that in ventilating topics incident to the medical and nursing professions you are actuated only by the purest of motives, I will not apologise for trespassing at such length.

[At the conclusion of our Notes we distinctly emphasised the difficulties of the situation, and did not in any way blame the matron.—Ed. The Hospital.]

### APPLICATIONS FOR UNCERTIFICATED NURSES.

"Hard to Convince" writes: A newspaper report has been sent me which states that Miss Lückes, in her evidence before the Select Committee of the House of Commons, declared that she had as many applications for uncertificated nurses as for those certificated. I wonder if Miss Lückes really said and meant this. I have known many instances where nurses, a long way from the end of their training, have been sent out to private cases, but I have also understood that it would be a great concession on the part of a hospital committee to allow a nurse under these circumstances to attend even her own friends though they might wish particularly for her services, and would, of course, pay for them in the usual way. To send out a nurse who, with the kindest heart or steadiest head may, through lack of experience, allow a patient to run any risk is, to my mind, manifestly unfair to all concerned—the public, the nurse, and the institution. There is, I imagine, no sliding scale in hospital fees; therefore the public would be paying for qualified services which it might not get. The nurse might be stamped as inefficient before her career had really begun and the institution might get a name for producing badly-trained nurses. I know only too well there are women in the profession whom not all the training in the world would make ideal nurses, but the "hall mark" of registration would at least guarantee a certain amount of head work which might in a crisis be all that would be necessary. Again, professional nurses do not, as a rule, take permanent cases. The fees are weekly, the engagement presumably the same. With this understanding an undesirable person may be quickly and easily disposed of, and a nurse—not that I admit she would desert a patient for any but grave reasons—may quite as easily dispose of an undesirable case. I should like to know what other people think of the demand for uncertificated nurses.

### MR. SYDNEY HOLLAND AND REGISTRATION.

"Onlooker" writes: I would certainly write to Mr. Holland if I had time, and hope that in the future I may have the pleasure of knowing him. I am aware that in the past he has done good work for the London and Poplar Hospitals, as also for patients and the nurses. I wish he would alter his opinion about State registration. I think that in time it will be passed, as in the United States, in spite of much opposition. Union is strength, and that is what is needed, and especially for our largest training schools, to take the lead in this question, instead of finding after the struggle is over that they will have to accept it whether they like it or not. But while we are considering the question let us have the highest ideal that we can, more charity towards each other, more give-and-take, and certainly more loyalty towards each other, our patients, and our training schools. I do not agree with Mr. Holland's view that there should be at the head of a "Nurses' Directory" the following sentence "No nurse by being in this Directory is guaranteed to be a good nurse." I am afraid that Mr. Holland has met with more black sheep in the profession than I have. We know that there are many but they are not all so black as they were painted by a certain lady in the *Nineteenth Century* of 1897, and I think that if Mr. Holland had been in as many households as I have, in my capacity of private nurse, that he would find that the public of to-day are somewhat different to the public of 15, or even 10 years ago. They know more about nurses and institutions, and they are not at all likely to be deluded by the short-sighted, stupid, or even worse, nurse.

### A NOVEL PROPOSAL.

"K. A. C." writes: May I suggest that all nurses working on their own account should have their qualifications plainly stated on their cards thus:—Nurse (or Miss) Jones, 3 years' certificate Bristol General Hospital, 1 year Fever Metropolitan Asylums Board; or Nurse (or Mrs. Smith) Maternity Nurse, Queen Charlotte's Central Midwives Board. The date of training would give the amount of experience and also facilitate reference to the hospital named should a doctor doubt the statement. I do not mind who wears uniform, for I recognise that it is suitable to all engaged in institutions, also to workers amongst the poor, but I do object to women trained in maternity work only, and others, taking work under false pretences.

"Nurse Willett" writes: I was pleased to see the question of uniform taken up in your pages by a Trained Nurse. We feel very jealous regarding the uniform, and particularly so when we read, as some of us did in the daily papers, that in the neighbourhood of Marylebone parlour-maids, kitchen-maids, and general servants are donning the uniform and passing as trained nurses. We feel anxious that something should be done in order that the public may be able to tell who are nurses. Even nurse-maids are trying to copy the uniform. In one house I nursed in the nurse-maid wore a Dora cap and strings, which I too happened to wear. Surely a small brooch, or button with some crest or letters on could be arranged, and sold only on production of our certificate. I hope that this matter will be taken up by other nurses.

"A Lover of the Profession" writes: What a pity it is that members of the nursing profession continue to abuse one another over outdoor uniform. It is certainly very unpleasant to a trained nurse, and one who tries to uphold her profession, to see nurses in uniform untidily dressed, but surely no trained nurse worthy of the name would correspond to Nurse E. Woodburn Heron's description. I was trained in the South of London and have been at two institutions since, and in each place the matron was most particular about nurses being tidy and spotlessly clean both on and off duty. Whenever I see a nurse with bows too large and dresses too long, etc., I at once come to the conclusion she is untrained or a nursemaid. It would, indeed, be a splendid thing if uniform could be worn only by trained nurses, whose right it is (and only theirs), but may I venture to say that if nurses of to-day gave more thought to their work, and how the life of each patient she comes in contact with is influenced either for good or evil by her actions than to outdoor uniform, nurses would be better thought of by the public than they now are.

"Margaret" writes: As a "trained" nurse, may I say that I entirely agree with Nurse Woodburn-Heron's remarks on what may, for want of a better term, be called "stage" uniform. But let us put the blame where it belongs—*i.e.* on the shoulders of the superintendent of the nurse-training and nurse-employing institutions throughout the country. The wearers of this particular brand of uniform must be probationers. For surely no matron would retain on her staff a fully-trained nurse, whose term of training had left her with so little common sense. As a probationer, I myself wore a long-trained dress because I thought that it looked nice, and added to the dignity of my appearance; and I wore my apron under my cloak because it saved me the trouble of taking off and putting on. I knew nothing of germs and antiseptics practically. But my matron pounced upon my "little ways" and explained. I learned antiseptic principles as they apply to the nurse's person, and have never forgotten them. Has any one ever objected to trained maternity nurses wearing uniform? If so, maternity nurses should combine and carry the war into the enemy's camp, by adopting a special uniform of their own, grey for choice. In a very few years grey uniform would be looked upon as so much the maternity nurses' property that it would be misleading for ordinary nurses to wear it.

Sister L. S. G. writes: Although a fully-trained nurse, I sympathise with Nurse E. Woodburn-Heron's letter suggesting a reform in our own uniform before finding fault with our less fortunate sisters (as far as training is concerned). We hear much of the injustice of untrained women—nurse-maids, for instance—wearing our uniform, and it would puzzle the most discriminating to detect a trained hospital nurse; but no one can mistake a well-trained nurse. Neatness, cleanliness in every detail, spotless collar, cuffs, and strings (the latter to be tied in a bow of reasonable proportions and worn under the chin, not embracing the left ear), go to form a consistent *tout ensemble*. The absence of the apron, a dress which does not touch the ground by two inches all round and has no ragged edges, the well-brushed and tidily-dressed hair, go to prove that the nurse is acquainted with the elementary rules of hygiene, that she approves of them in the way she orders her own dress, and that she will bring her knowledge to aid her in the care of her patients. I have often wondered to what especial end the veil on bonnets, which so many nurses affect, is used? To me it seems to be utterly useless and the reverse of becoming. I think that jewellery with uniform is on a par with a rustling silk skirt under a cotton or cloth uniform dress. If nurses would only give themselves time to think what the profession is, they by their uniform represent, they would discard or amend those things or faults which are likely to elicit the adverse criticism of the public, to say nothing of the opinion of the nursing world.

### Novelties for Nurses.

(By Our Shopping Correspondent.)

### A CHARMING PICTURE.

Messrs. Bovril have sent to the Editor a charming engraving entitled "Little Lady Bountiful," after Fred Morgan's picture. This engraving is offered to purchasers of Bovril who send coupons of a face value of one guinea and sixpence for postage. The coupons accompany every jar, tin, or bottle of Bovril. The offer is open until June 1905. The subject is a rural picnic. A pretty group of the children of the rich is assembled under a tree by the banks of the river. Little Lady Bountiful is depicted presenting some of the out-spread feast to two small barefooted urchins who have been attracted to the spot. The scene is a happy picture of English life.

### FURNISHING A BED-SITTING-ROOM.

Many nurses are obliged, owing to want of room, to practise a little harmless deception in the matter of furniture, as, for example, endeavouring, by means of cushions and a pretty coverlet, to make a bed look as much as possible like a sofa or cosy corner, while even the unsightly washstand is sometimes more or less disguised. Messrs. Oetzmann, realising the difficulty of economising space, and at the same time the growing taste for artistic effect, have evolved a suite of furniture which, without any troublesome transformation, does equally well for a bedroom by night and a sitting-room by day, ,and I should advise those about to furnish or to replenish the fittings of a room of this kind to send for a descriptive and illustrated account of the "Chameleon" suite, or, still better, to pay a visit to 67—79a Hampstead Road, W., where it is on view. There are four pieces of furniture in the suite, a bed which is also a settee, a washstand which turns into a cabinet, a cheval glass which no one will object to seeing in a sitting-room, and a hanging press which has every appearance of being a bookcase. The bed is 2 feet 6 inches wide and 6 feet 6 inches in length, both ends having a row of upright laths of equal height. There is a wire-wove mattress, and underneath this is a rack upon which the bedclothes may be neatly folded and put away by day; they are hidden by a valance, and the mattress and pillows being covered with the same the whole effect is very pleasing. To complete the settee a wooden and upholstered back runs from end to end; this can be removed at night and put back again in the morning, or whenever the settee is required. The covering of both mattress and cushions slips off, and these latter become pillows. The accompanying illustration shows the washstand open; when closed, and a flower or plant placed upon it, it makes an artistic cabinet to which square glazed doors give a quaint finish. A cheval glass which forms part of this suite has a square cupboard, and this could be used for a low table, or a palm could be stood upon it in front of the glass. The height of the glass is 5 feet 6 inches, and it can be fixed in its frame or tilted as required. The hanging wardrobe, with the motto "To maken Virtue of necessity," copper fittings, and leaded glass, through which the contents are invisible, and the ingeniously contrived space below, with a curtain to draw, to take a bandbox, boots, or other small things, is a very handsome piece of furniture, and indeed the set is decidedly well worth considering as a solution of the difficulty above alluded to. The pieces are made in fumigated oak, which can be graduated in depth of tone, and has a quiet, restful look. Here are the prices:—Cabinet and washstand, £5 17s. 6d.; mirror, £2 18s. 6d.; cabinet (or bookcase) and wardrobe, £5 5s.; bedstead and settee, £4 17s. The entire suite costs 18 guineas, while to complete the room there are chairs and tables in corresponding style, and a wall cupboard with copper hinges for 3s. 9d.

### Appointments.

[No charge is made for announcements under this head, and we are always glad to receive, and publish, appointments. The information, to insure accuracy, should be sent from the nurses themselves, and we cannot undertake to correct official announcements which may happen to be inaccurate. It is essential that in all cases the school of training should be given.]

Beckett Hospital, Barnsley.—Miss S. Mathers and Miss A. Galloway have been appointed sisters. Miss Mathers was trained at the Royal Victoria Hospital, Belfast, where she was temporarily sister. Miss Galloway was trained at Rochdale Infirmary, where she has since been night sister. She has also been staff nurse at the Royal Infirmary, Sheffield.

Birmingham Ear and Throat Hospital.—Miss Edith Bone has been appointed sister. She was trained at Hull Royal Infirmary.

Borough Sanatorium, Bridlington.—Miss Mabel Garrood has been appointed nurse in charge. She was trained at Northampton Borough Hospital, and has since been nurse at Leicester and Croydon Borough Hospitals, and charge nurse at Isolation Hospital, Hertford.

Cottage Hospital, Fordingbridge, Hants.—Miss Agatha Laughlin has been appointed matron. She was trained at the Royal Free Hospital, Gray's Inn Road, London. She has since been sister at the Southwark Infirmary, the Royal Victoria Hospital, Belfast, the Royal South Hants Hospital, Southampton, and night superintendent at Chichester Infirmary.

Evesham Union Infirmary.—Miss Sarah Alice Phillips has been appointed head nurse. She was trained at the

Birmingham Workhouse Infirmary, and has since been assistant nurse at the Cheltenham Union Infirmary. She is registered under the Central Midwives Board.

HOUSE OF RECOVERY AND FEVER HOSPITAL, CORK STREET, DUBLIN.—Miss Augusta Minshull has been appointed assistant matron. She was trained at Chester Infirmary, where she has since been day sister and night superintendent.

LEAVESDEN ASYLUM.—Miss Lucy C. Cooper has been appointed superintendent nurse of female blocks. She was trained at the Central Sick Asylum, Hendon, and has since been nurse at the Isle of Thanet Infirmary, Kent. Before receiving her general training she was trained at the Brighton branch of the Holloway Sanatorium for the Insane, and was for three years and a-half in the lunatic wards of the Brighton Infirmary.

MINERS' HOSPITAL, REDRUTH.—Miss Edith Riley has been appointed charge nurse. She was trained at Salford Union Infirmary, Pendleton, near Manchester, and has since been assistant nurse and charge nurse at Salford County Hospital, Manchester.

RIPON DISPENSARY AND COTTAGE HOSPITAL.—Miss A. C. Lacy has been appointed nurse-matron. She was trained by the nursing sisters of St. John the Divine and at the Walsall and District Hospital, at which institution she has since been charge nurse and night sister.

ST. MARK'S HOSPITAL, CITY ROAD, LONDON.—Miss Fanny L. Mason has been appointed night sister. She was trained at the Royal United Hospital, Bath, where she has since been in charge of the medical and surgical out-patients' and casualty department.

ST. NEOT'S WORKHOUSE INFIRMARY.—Miss A. E. Bond has been appointed charge nurse. She was trained at Aston Union Infirmary, Birmingham, and was afterwards nurse at the Tendring Union Infirmary.

THE RETREAT, YORK.—Miss Ethel Howard has been appointed night superintendent. She was trained at Cheddleton Asylum, Leek, where she has since been sister.

UNION INFIRMARY, WARRINGTON.—Miss Lucy Alice Dawes has been appointed night sister. She was trained at the Union Infirmary, Selly Oak, and holds the certificate of the Central Midwives Board.

UNION INFIRMARY, WOLVERHAMPTON.—Miss Sarah Atkinson has been appointed sister. She was trained at the Union Infirmary, Selly Oak, and holds the certificate of the Central Midwives Board.

### TRAVEL NOTES AND QUERIES.

BY OUR TRAVEL CORRESPONDENT.

A HOLIDAY IN THE TYROL (Robin).—In choosing to spend your holiday in the Tyrol I fear you have fixed upon a very expensive, though delightful, trip. I think you can just manage on the £15 each, if you stay only at Innsbrück and Cortina; the latter is an exquisite spot in the mountainous part of Tyrol. Second-class return to Innsbrück, *viâ* the Arlberg, is £6 13s. 2d. Travel straight through to Zurich, where you must sleep. Go to Hôtel St. Gothard, in the Bahnhof Strasse. Ask for rooms on the third or fourth floor always. By making an early start the next morning I think you might reach Innsbrück without sleeping again on the way. At Innsbrück go to Carl Kayser's Pension Hotel. You must stay three days if possible. The situation of Innsbrück is ideal, and the excursions endless. As you cannot make many moves, each one adding so much to cost, I should advise your spending the rest of the time at Cortina. To reach this take train over the Brenner to Sterzing, where I think you must sleep. Go to Hôtel Neue Post. The next day take train to Toblach, where a diligence leaves daily for Cortina. Almost certainly you must sleep at Toblach. Go to Hôtel Ampezzo. At Cortina go to Hôtel Zum Weiszen Kreuz. You see it will have taken you six days; but your return may occupy less time, because you will already have seen Innsbrück. I cannot tell you the return fare between Innsbrück and Cortina d'Ampezzo, but it is a long distance, and will, I fear, be quite £3. Remember, too, that the money and rate of exchange is not very favourable to us, and one does not gain as in Italian countries. If I can help further let me know. I fear it is a difficult tour for inexperienced travellers whose purses are slender, and the time you have at your disposal is short for so distant a trip.

JOURNEY TO CANADA (S. S.).—I cannot read your pseudonym. Send a stamped and addressed envelope to Messrs. Cook, Ludgate Circus, E.C., and ask them to quote you the lowest prices of tickets to Canada and the dates of the sailing of the vessels. As to clothes you will want them very warm. Canada is intensely cold in the winter. Have warm underclothing and good, strong boots. You can adopt any uniform you like, but always remember that it is necessary to have really warm clothes.

NEAR LYNTON AND LYNMOUTH (Tudor).—I fear you will find nothing approaching in cheapness to the terms you mention. I do not think there is anything to be had there under 25s. to 35s. per week. It is a very expensive locality. I should be pleased to try and help you, but I know there is nothing to be had of the kind you need.

THE BAY OF ST. MALO (Nurse Rose).—I think St. Servan or St. Malo will suit you well, but farmhouse lodgings do not exist in France. I do not think you will get anything quite so cheap as you desire. When do you want to go?—season makes so much difference in price. The cheapest place I know is near to St. Malo, but it comes to £1 8s. per week. If this will do I think the situation would suit you, as it is so near to Dinard and Dinan —both lively places. If this will not suit let me hear again and I will suggest something else.

BRUGES, ANTWERP, AND BRUSSELS (Old China).—If you can manage for yourselves I do not advise hotel coupons because it is not necessary to pay at so high a rate and I am not a friend to tours planned by companies, because you must go just where you are told and nowhere else, regardless of individual taste; if I knew what time you have at your disposal and how much you wish to spend I could help you better. Write to me again with all particulars. In the meantime, think out this route. London to Ostend, only 6s. second class, by G.S.N.C.; three days at Bruges (a fascinating place); two at Ghent, from whence visit Courtrai and Oudenard and Alost; four days at Brussels, from whence see Louvain and Mechlin; three or four days at Antwerp, and home by G.S.N.C. You need not spend more than 8 frs., which is 6s. 8d. daily at hotels, even counting tips. Belgian railways are very cheap. I have no experience of the circular tickets bought in the country for short tours. I never use them except on long journeys. Tell me what you think of this plan and anything else you want to know. I will then give you addresses.

DEVON, SCOTLAND, OR BRITTANY (Tares).—I am pleased to help and it is no trouble, but if economy is essential, the two first countries will not do at all, and the sum you mention would not cover three weeks, much less a month. Nothing could be nicer than Brittany for three weeks, or what do you think of Belgium? See answer to "Old China." These are the only two places where the sum at your disposal would cover three weeks. You must not think of Devon or Scotland. If you go to Brittany, as I understand, you go with a brother and he chooses the route, if not let me hear again and I will plan you a little tour for three weeks to come within the limit you speak of.

RULES IN REGARD TO CORRESPONDENCE FOR THIS SECTION.—All questioners must use a pseudonym for publication, but the communication must also bear the writer's own name and address as well, which will be regarded as confidential. All such communications to be addressed "Travel Correspondent, 28 Southampton Street, Strand." No charge will be made for inserting and answering questions in the inquiry column, and all will be answered in rotation as space permits. If an answer by letter is required, a stamped and addressed envelope must be enclosed, together with 2s. 6d., which fee will be devoted to the objects of "The Hospital" Convalescent Fund. Ten days must be allowed before an answer can be published.

### Notes and Queries.

**For REGULATIONS see page 70.**

*Taking Temperatures.*

(33) Will you tell me where I can be taught to take temperatures and to fill in charts?—*E. M. H.*

In any hospital.

*Nursing Without Examination.*

(34) Can you tell me where I could learn nursing without going in for an examination, and where a salary is given?—*Anxious.*

A nursing home might do as you desire, but we fear that the knowledge you would acquire would not be of much use to you afterwards. You had better advertise.

*Nurses and Hypodermic Injections.*

(35) Will you tell me whether a doctor has a right to expect a nurse to give hypodermic injections of morphia. I have never been allowed to do so in hospital but the doctor here says that I cannot be "fully trained" unless I can do so.—*Rallying.*

A nurse should be prepared to give hypodermic injections if requested to do so by the medical attendant.

*Books Required.*

(36) Can you tell me of a good book of cookery recipes for persons suffering from diabetes?—*L. H.*

"The Art of Feeding the Invalid," published by the Scientific Press.

Can you recommend me a book on cottage hospital management and housekeeping?—*Nurse-Matron.*

"Cottage Hospitals." By Sir Henry Burdett. 10s. 6d. post free from the Scientific Press Co., 28 Southampton Street, Strand, W.C.

*Midwifery Certificate.*

(37) I am a National Health student and wish to get the L.O.S. certificate as well. To whom shall I apply?—*Horley.*

The London Obstetrical Society has ceased to exist. Under the new Act, certificates are issued only by the Central Midwives Board. Write to the Secretary, 6 Suffolk Street, Pall Mall, S.W.

*Military Nursing.*

(38) Will you give me some information about military nursing?—*A. M. I.*

All military nursing is now carried out under the auspices of Queen Alexandra's Imperial Military Nursing Service. Details can be obtained from the Under-Secretary of State, War Office, Pall Mall, S.W.

*Training School.*

(39) Can you tell me anything as to the training at the Royal Infirmary, Newcastle-on-Tyne?—*Emma.*

The training is for three years, and the certificate should qualify you to apply for any appointment afterwards. If you cannot gain admission consult "The Nursing Profession: How and Where to Train." You will find details there of all the hospitals.

*The Wearing of Uniform.*

(40) I have been nursing as an attendant for 12 years, and my present patient wishes me to wear uniform. Is it right for me to do so, not having been trained?—*Uniform.*

There is no law as to the wearing of uniform, and you may do so if you wish.

*Sanatorium Probationers.*

(41) Do Fresh Air Sanatoria train probationers or do they prefer them trained in a general hospital?—*C. S.*

Most of these institutions train their own probationers. Either advertise or write to the matrons of the principal sanatoria. You can find them in the list published by the National Association for the Prevention of Tuberculosis and obtainable from the Scientific Press, 28 and 29 Southampton Street, Strand. Price 7d. with postage.

*Nursing Hernia.*

(42) Can you tell me of any numbers of The Hospital, or recommend me a book, to help me nurse a patient to be operated on for hernia, "radical cure?"—*Nurse Janet.*

Consult a nursing text-book.

**Handbooks for Nurses.**

| | Post Free. |
|---|---|
| "How to Become a Nurse: How and Where to Train." | 2s. 4d. |
| "The Nurses' Dictionary" (Pronouncing) ... ... | 2s. 0d. |
| "Nursing: its Theory and Practice" (Lewis.) ... ... | 3s. 6d. |
| "The Light Treatment" (just published) ... ... | 2s. 6d. |
| "A Complete Handbook of Midwifery." (Watson.) ... | 6s. 4d. |

Of all booksellers or of the Scientific Press, Limited, 28 & 29 Southampton Street, Strand, London, W.C.

### For Reading to the Sick.

"AS YEAR BY YEAR."

"As year by year the thrills of spring-tide shoot,
Through earth's dull veins with fresh magnetic might,
Nor fail, for frosts that nip and winds that blight,
To draw from bud to bloom, from bloom to fruit;
So, Lord, who erst didst stir with quickening power
My answering soul, achieve what Thou hast aimed;
Draw, for Thou hast drawn; hold, what Thou hast claimed;

"Draw through all failure to the perfect flower;
Draw through all darkness to the perfect light.
Yea, let the rapture of Thy spring-tide thrill
Through me, beyond me, till its ardour fill
The ungrowing souls that know not Thee aright.
That Thy great love may make of me, even me,
One added link to bind the world to Thee."

*E. S. A.*

We must not be perplexed or put out if we have to change our plans. God sends us hither and thither; we may think that we are wasting our special talents, when God has, after all, some particular need for our particular work at a particular time. And equally we must learn to measure our strength; we cannot all do the same things, we are not all adapted to the same work, or charged with the same duties. Why should we overstrain ourselves in that which is beyond our strength, or neglect plain duties for others less obvious?—*Canon Newbolt.*

The trials, whether of body or of spirit, which gradually wear our life away, are God's own chosen means of fulfilling what we have already professed to be our true vocation. They are the necessary accomplishment of that sentence on the flesh which, ever since we knew Christ, has been the object of our fondest desire that, dying with Him, we may rise with Him; that, through the daily Cross, the daily resurrection may be ours; that "the sentence of death" being in us, the power of the Divine life may triumph in us. Shall we murmur at these strokes, which are verily the unloosening of the bonds to set the captive spirit free? Shall we regret the gradual decay, which is but the passing off of the gloom of the night before the dawn of the everlasting Day? Would we check the progress of our dissolution, if we could? All that bows the vigour of our fleshly frame, all that consumes away the spirits and strength of the sensitive soul, are in truth but the travail-pangs of the perfected deliverance of the Divine Nature struggling within us, for its heavenly development. Be not, then, unwilling to yield up the flesh to this slowly-advancing death, which must increase until its end be accomplished, "until the day break, and the shadows flee away."—*Rev. T. T. Carter.*

Oh, ask not thou, How shall I bear
The burden of to-morrow?
Sufficient for to-day, its care,
Its evil and its sorrow;
God imparteth by the way
Strength sufficient for the day.

*J. E. Saxby.*

## Figures and Tables

**Figure f1:**
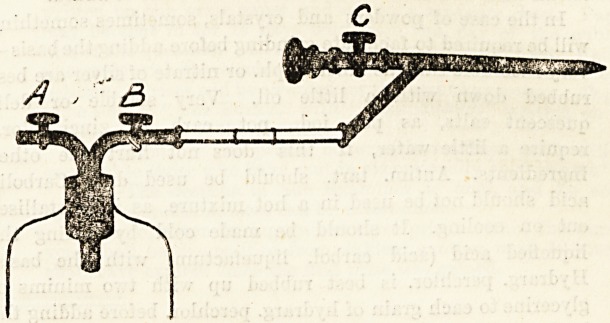


**Figure f2:**
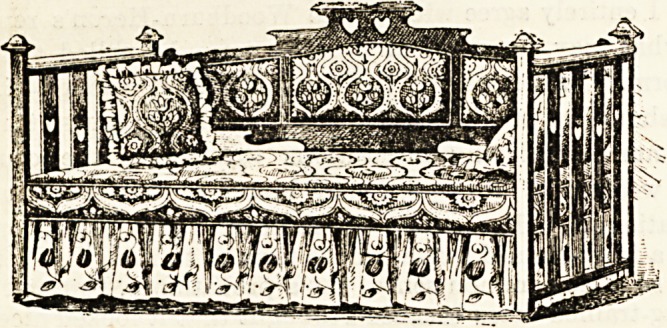


**Figure f3:**